# A Survey of Hospital Optometrists Performing Ophthalmic Laser Procedures in the United Kingdom

**DOI:** 10.1007/s44402-026-00107-2

**Published:** 2026-05-19

**Authors:** David Grant Robinson, Deacon Harle

**Affiliations:** School of Optometry and Vision Sciences, Cardiff University, Cardiff, UK

**Keywords:** Healthcare surveys, Lasers, Ophthalmology, Optometrists, Public health

## Abstract

**Introduction:**

In the United Kingdom (UK), the role of optometry has advanced beyond sight testing to the delivery of clinical and medical treatment of the eye. Over the past decade, optometrists have become increasingly involved in ophthalmic laser treatments. To better understand the scope, autonomy and training requirements of optometrists involved in laser provision, this study sought to survey optometrists performing laser treatment in the hospital eye service.

**Methods:**

This study was a descriptive, cross-sectional survey of hospital optometrists conducting laser procedures in the UK. During a 6-week period from May to June 2025, an electronic survey was disseminated to all hospital optometrists who had opted to be included on a centralised UK database and via established professional communication channels. The survey was divided into two sections. The first section collected demographic data while the second section explored application and training.

**Results:**

Seventy-five hospital optometrists responded to the survey. The typical amount of time per week (mean ± standard deviation) performing laser procedures was 5 h (±2). Over 90% reported they work independently and autonomously when performing laser procedures. The most commonly performed procedures were Nd:YAG capsulotomy, selective laser trabeculoplasty and peripheral laser iridotomy. Ninety per cent of respondents had undertaken internal training. A significant association was found between the number of years qualified and duration of performing laser procedures (Fisher’s exact test, *p* < 0.01). No independent factors relating to the scope of practice or autonomy were identified via multivariable logistic regression (*p* > 0.05).

**Conclusion:**

This study strongly suggests that hospital optometrists perform ophthalmic laser procedures autonomously across the UK. Activity predominantly relates to cataract and glaucoma care. Within these areas, hospital optometrists readily conduct laser procedures such as Nd:YAG capsulotomy and selective laser trabeculoplasty. The results of this study suggest that, presently, national clinical guidelines do not reflect this autonomy and should be amended accordingly.

Key Points

- Hospital optometrists are involved in the delivery of ophthalmic laser procedures across the United Kingdom. Activity predominantly relates to glaucoma and cataract care. Training is predominantly conducted in-house.
- Hospital optometrists readily conduct ophthalmic laser procedures such as selective laser trabeculoplasty and Nd:YAG capsulotomy independently and autonomously, making the clinical decision to treat themselves.
- National clinical guidelines relating to ophthalmic laser provision by optometrists do not reflect this autonomy and should be amended accordingly.

## Introduction

Optometry is a healthcare profession that is autonomous, educated and regulated [1]. In the UK, the optometrist’s role continues to evolve beyond the testing of sight. This trajectory was established in 1999 following changes to the General Optical Council (GOC) regulatory rules around referral [2]. This resulted in optometrists taking a more active role in the treatment of ocular abnormality, injury and disease, as opposed to referring all ocular pathology cases to a medical practitioner. Alongside this change, the 1999 Crown Report recommended that optometrists be amongst the first group of professionals outside medicine and dentistry to become non-medical prescribers [3]. This action was facilitated 6 years later by a further GOC amendment that empowered optometrists to offer medical and clinical treatments more readily to their patients [4]. Ultimately, this legislation has led to the opportunity for optometrists to gain specialty registration and regulation as independent, non-medical prescribers, with the capability to treat the eye and ocular adnexa [5, 6].

In the main, optometric clinical treatment has focused on the use of medicines, both topically and orally. However, not all ocular disease is best managed in this way, with some treatments requiring other approaches, including the use of ophthalmic lasers. Although lasers have been used in eye care for many years, recent developments relating to the management of ocular hypertension (OHT) and chronic open-angle glaucoma (COAG) are likely to result in the increased use of selective laser trabeculoplasty (SLT) [7]. This, in turn, could facilitate the involvement of optometrists in the delivery of SLT to meet demand.

In the UK, changes to optometrist’s scope of practice over the past few decades have resulted in improved access to eye care services and a reduction of the burden on a stretched ophthalmology service [8]. With significant workforce demands, optometry and ophthalmology have successfully worked together to deliver a wide range of clinical services to patients. The Clinical Council for Eye Health Commissioning (CCEHC) is an example of a collaborative initiative designed to provide evidence-based national clinical leadership, advice and guidance to policy makers in health, social care and public health in England [9]. The CCEHC designates eye care under three commissioning banners: primary eye care, community services (previously called community ophthalmology) and the hospital eye service (HES). Optometrists in the UK work in each of these settings. The majority of UK optometrists work in National Health Service (NHS) primary eye care services (with different contractual arrangements in each of the four UK nations). Fewer work in community eye services, and a small number work in secondary care HESs [10–12].

The body of evidence underpinning hospital optometrist involvement in ophthalmic laser procedures such as SLT and Neodymium:Yttrium Aluminium Garnet (Nd:YAG) capsulotomy is growing [13–18]. The latest workforce surveys reported that in 2020, optometrists were regularly delivering SLT in 14 glaucoma units in the UK compared to one unit in 2016 [13, 14]. A large proportion (>80%) also reported that their practice within a hospital glaucoma care setting was predominantly independent [13]. More recently, a comparative study of SLT conducted in three UK hospitals (*n* = 207 eyes of 131 patients) found that 24-month outcomes, including change in visual acuity, intraocular pressure and glaucoma drops pre- to post-SLT, were comparable between optometrists and ophthalmologists [15]. This outcome aligned with evidence from Scotland 5 years prior that also reported a similar efficacy between optometrists and ophthalmologists within an SLT service [16].

The implementation of SLT clinics led by optometrists could help strengthen the UK glaucoma care service by increasing the opportunity to treat people with COAG and OHT promptly, while allowing ophthalmologists to prioritise more complex cases. However, the extent of optometrists’ clinical readiness to deliver such a service may be affected by previous training [19]. A current concern is that training within the HES to perform laser treatments remains local and ad-hoc, with the potential for inconsistencies [19, 20].

The UK Ophthalmology Alliance have published policies and procedures that hospitals training optometrists in ophthalmic laser procedures may adopt, but there is no obligation to do so [21]. In 2021, Northeastern State University, Oklahoma College of Optometry, USA, delivered an advanced procedures course that provided ophthalmic laser and surgical training to UK optometrists. Subsequently, Moorfields Education, in conjunction with the University College London Institute of Ophthalmology, developed a UK-based ophthalmic laser course designed for ophthalmic healthcare professionals [22]. More recently, other UK institutions have followed suit. Cardiff University has become the latest institution to offer training in this field, launching its ophthalmic therapeutic laser procedures course in 2024 [23]. Aston University also offers ophthalmic laser training to optometrists as part of a stand-alone advanced optometry and ophthalmology clinical skills workshop [24].

Both Moorfields Education and Cardiff University train optometrists to perform ophthalmic laser treatments with blended learning via lectures and tutorials, followed by practical face-to-face training using model eyes on three laser techniques: Nd:YAG capsulotomy, SLT and laser peripheral iridotomy. The Moorfields course defines further stages of training: structured in vivo training with competency sign-offs, building up a portfolio with formative assessments and case-based discussions, defined self-audit and reflective statements underpinned by medical education theory and principles [25]. In contrast, the Cardiff University course suggests trainees develop their ophthalmic laser skills further with post-course supervised training and experience in delivering therapeutic ophthalmic laser procedures.

Despite the range of postgraduate training available, there is no obligation for optometrists to undergo formal, university-based postgraduate training in ophthalmic laser. Local ad-hoc training within hospital eye departments without formal review is an acknowledged route to gain experiential learning. Against this varied training backdrop and the growing involvement in laser provision by hospital optometrists, the present study sought to improve understanding of the current landscape by exploring the demographics, scope of practice, level of autonomy and history of training of UK hospital optometrists who perform ophthalmic laser procedures.

## Methods

This study was a descriptive cross-sectional survey of hospital optometrists conducting laser procedures in the UK. The research adhered to the tenets of the Declaration of Helsinki and was reviewed by the Cardiff University School of Optometry and Vision Sciences Research Ethics Committee (reference SREC 1640). The survey was developed using an electronic platform (Microsoft MS Forms; Microsoft.com), which enabled digital design and distribution to respondents via an e-link or QR code. A digital invitation was disseminated to all hospital optometrists who opted to be included on a centralised database collated and maintained by the UK Hospital Optometrists Committee. To drive engagement, the survey was also distributed via established professional communication channels, including social media and digital messaging platforms, likely known to optometrists providing laser procedures.

The questionnaire (Supplement 1) was designed to be self-administered and anonymous, with an average completion time of approximately 3 min as established via a pilot study. It consisted mainly of multiple-choice, nominal questions. To permit comparison of change and enhance validity, the questionnaire was based on that used from existing published works [13, 14], adapted for the target audience. This included expansion into specific areas such as geographical location, duration of performing ophthalmic laser procedures and current scope of ophthalmic laser practice. Before progressing to the main body, participants were required to read a preamble. This provided an overview of the research, a link to a detailed participant information sheet and a statement of informed consent.

The main body had 18 questions divided across two sections. The first section collected demographics relating to the participants’ duration of qualification, location, work environment, activity and experience performing laser procedures. The second section focused on application and training. Questions explored what laser procedures they currently perform and their level of autonomy. Information relating to teaching and assessment during their training period was also collected. Data were initially stored on the platform’s secure server before being exported into the Statistical Package for Social Sciences (IBM SPSS Statistics for Windows version 26; ibm.com) for analysis. Descriptive statistics were primarily used to analyse the dataset in accordance with the objectives. As the sample size was small, formal comparisons of years qualified and years performing ophthalmic laser procedures were conducted using Fisher’s exact test. Multivariable logistical regression analysis was performed to identify potential independent factors of performance; this included multinomial and binary logistical regression strategies as appropriate. A threshold of *p* < 0.05 was taken as statistically significant.

To aid visualisation, publicly available software was used to generate a choropleth. A post hoc review of the complete dataset was conducted to scrutinise the validity of any duplicate or highly similar entries.

### Sample Size

As of November 2023, there were approximately 16,500 GOC-registered optometrists in the UK [12]. The vast majority (81%) were based in England. The number of registrants in Scotland, Wales and Northern Ireland were 10%, 5% and 4%, respectfully [12]. Presently, the exact number of hospital optometrists in the UK is unknown. Based on the second national survey of hospital optometrists conducted by Gunn et al., a reasonable estimate in 2020 was approximately 2–3 full-time equivalent (FTE) optometrists per unit [13]. Based on this, a conservative estimate of 2.5 optometrists per unit (estimate *n* = 130–150) suggested around 300–400 optometrists (~2% of all GOC-registered optometrists) working directly within the HES sector in 2020–2023.

In 2020, only 10–15% (*n* = 30–60) of HES optometrists were providing therapeutic laser procedures [13]. However, little is known about the expansion of hospital optometry over the past 5 years on a national scale. It is reasonable to suggest, as per the trend from 2016 to 2020, that the number of HES optometrists has continued to grow. Based on the continued rate of growth observed from 2016 to 2020, the population of hospital optometrists involved in laser procedures would likely be in the region of 90–100 in 2025 [13, 14]. Sample size calculation using this estimate and an online sample size calculator suggested that a statistically viable number of respondents for this survey would be 74–80 (population size estimated as 90–100, confidence level 95% and margin of error 5%.

## Results

The survey was live for a 6-week period from May to June 2025. During this time, 75 responses were received. This represented a response rate of 60% of the optometrists (*n* = 126) included on the Hospital Optometrists Committee distribution list, which was the primary method of distribution. However, the true response rate is unclear due to the use of other methods of dissemination and the consideration that not all UK-based hospital optometrists are listed on the Hospital Optometrists Committee database. All respondents answered all questions and were included in the analysis.

### Demographics

**Fig. 1:**
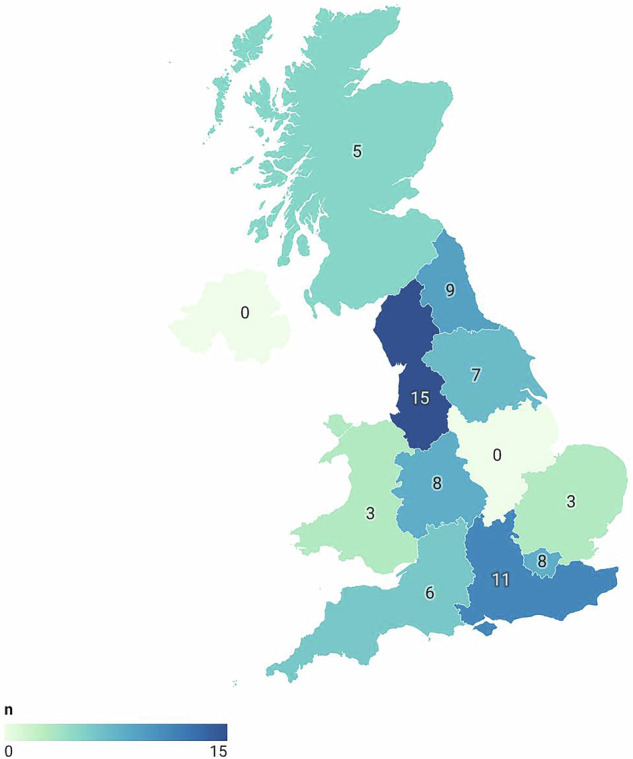
Number and location of respondents from across the UK. Darker shades represent areas from which more responses were received (as opposed to the overall optometrist workforce distribution). Graphic created using publicly available software (Datawrapper GmbH, datawrapper.de).

An overview of the location of respondents is given in Fig. 1. The vast majority of responses (89%) were from optometrists based in England. Three (4%) and five (7%) responses were from Wales and Scotland, respectively. No responses were received from Northern Ireland. Regional differences existed within England. The highest number of respondents (*n* = 15) was from the North West region, followed by the South East (*n* = 11) and North East (*n* = 9) regions. The only region in England without representation was the East Midlands.

Those who responded reported an average (±SD) of 30 (±10) h per week as a hospital optometrist. This equates to 0.80 FTE based on a typical 37.5 h week as an NHS employee. The average number of hours per week (±SD) dedicated to laser eye procedures was 5 (±2), equating to 0.15 FTE. There was a prevalence for respondents to dedicate either half a day (equating to 3–4 h per week, *n* = 32) or a full day (equating to 7–8 h per week, *n* = 26) per week to performing laser procedures. The range of hours dedicated to laser provision was 12 (lowest 2, highest 14 h per week). Two respondents engaged only in laser procedures as their hospital optometrist role, each working 1 day only.

### Involvement in Ophthalmic Laser Procedures

A summary of years qualified and involvement in laser ophthalmic procedures is provided in Table 1. The largest proportion of respondents (*n* = 20, 27%) had been qualified for over 20 years. Comparatively, similar numbers were found to be qualified within the 0–5 (*n* = 15, 20%), 11–15 (*n* = 15, 20%) and 16–20 (*n* = 14, 19%) year ranges. Those within the 6–10 years qualified range represented the lowest proportion of respondents (*n* = 10, 14%). No group included a number of respondents (*n* ± SD) outside two standard deviations of the mean across all groups (14.8 ± 3.6).

**Table 1:** Summary of years qualified as an optometrist and years performing ophthalmic laser procedures.

		Years qualified as an optometrist					
		0–5	6–10	11–15	16–20	20+	Total
Years performing laser *n* (%)	<1	10 (67)	3 (30)	1 (6)	1 (7)	2 (10)	17 (23)
	2–3	5 (33)	7 (70)	7 (47)	6 (43)	6 (28)	31 (41)
	4–5	0 (0)	0 (0)	3 (20)	4 (29)	2 (10)	9 (12)
	5–10	0 (0)	0 (0)	4 (27)	3 (21)	7 (32)	14 (19)
	10+	0 (0)	0 (0)	0 (0)	0 (0)	4 (20)	4 (5)
	Total	15 (20)	10 (14)	15 (20)	14 (19)	21 (27)	75 (100)

A significant association was found between the number of years qualified and duration of performing laser procedures (Fisher’s exact test, *p* < 0.01). There was a tendency for practitioners who had been qualified longer to have also been involved in laser provision for a greater period of time. Approximately a quarter of respondents had been performing laser procedures for less than a year; the majority of these (67%) were newly qualified (0–5 years as a fully registered optometrist). Conversely, only four (5%) had been performing laser procedures for over a decade. The most common timescale for involvement across all respondents was 2–3 years (41%). These findings highlight the recent upregulation of involvement in laser ophthalmic procedures by hospital optometrists.

Table 2 provides an overview of procedures currently performed and those likely to be performed in the near future. The most commonly performed procedures (n, % of respondents) were Nd:YAG capsulotomy (66, 88%), SLT (29, 39%) and peripheral laser iridotomy (15, 20%). Less common but still performed by hospital optometrists were laser iridoplasty (3, 4%) and laser vitreolysis (2, 3%). SLT was cited as the procedure not done presently but most likely to be performed in the near future (22, 29%). Second was peripheral laser iridotomy (16, 22%), followed by laser iridoplasty and laser retinopexy (both 3, 4%).

**Table 2:** Summary of ophthalmic laser procedures performed now and likely to be performed in the near future.

	Perform now (*n*, %)	Perform in the near future (*n*, %)
Nd:YAG capsulotomy	66 (88%)	2 (3%)
Selective laser trabeculoplasty	29 (39%)	22 (29%)
Peripheral laser iridotomy	15 (20%)	16 (22%)
Laser iridoplasty	3 (4%)	3 (4%)
Laser vitreolysis	2 (3%)	2 (3%)
Laser retinopexy	1 (1%)	3 (4%)
Pan retinal photocoagulation	1 (1%)	2 (3%)
Focal and grid macular laser treatment	1 (1%)	2 (3%)

Percentage values denote the proportion of total respondents (*n* = 75 total) who reported they currently perform, or will shortly perform, a technique. Techniques not shown in the table because they received a low number of responses (≤1% total respondents) for both being performed now and in the near future included: small incision lenticule extraction, argon laser trabeculoplasty, endoscopic cytophotocoagulation, micropulse laser trabeculoplasty, femtolaser assisted cataract surgery, photorefractive keratectomy, laser in situ keratomileusis and femtosecond laser in cornea. Nd:YAG Neodymium:Yttrium Aluminium Garnet.

### Independent Prescribing, Autonomy and Training

In the UK, optometrists can supply and administer specified medicines required during and after ophthalmic laser procedures via patient group directions or if registered as an independent prescriber [5, 6]. In this survey, two-thirds of hospital optometrists (*n* = 50) providing laser eye procedures were also independent prescribers.

When asked about the level of autonomy, 91% reported that they work independently and autonomously, making the clinical decision to treat. A small proportion (*n* = 3, 4%) perform YAG capsulotomy independently; however, they sought the opinion of a consultant ophthalmologist prior to performing SLT. Only one optometrist conducted ophthalmic laser procedures under direct supervision; the supervisor being an independent prescribing optometrist.

All but two optometrists (97%) undertook their laser training in the UK. Both optometrists who were not trained in the UK had completed a programme of study at Northeastern State University, Oklahoma College of Optometry, USA. The vast majority of optometrists (80%) had only undertaken in-house training within their hospital. Far fewer had received training either from a higher education institution (7%) or from an independent provider not associated with a higher education institution (3%). Eight optometrists (10%) reported they had received both in-house hospital training and training from a higher education institution. During their training period, only one in four practised performing ophthalmic laser on simulated eyes.

Multiple benchmarks were used to denote the end of a training period. Most commonly, optometrists had to treat a predefined number of patient cases (85%) and/or demonstrate their clinical skillset in a non-formal setting (65%). Less commonly used assessment methods included having to (i) observe a predefined number of patient cases (55%), (ii) pass a theory assessment (35%) and (iii) pass a formal practical assessment (23%).

Multinomial and binary logistic regression were conducted to determine the effect of all other parameters investigated on the scope of ophthalmic laser practice and level of autonomy. The fit of each model was found to be significant (scope of practice, *χ*^2^ = 41.05, *p* < 0.01, pseudo *R*^2^ = 0.47; autonomy, *χ*^2^ = 31.19, *p* = 0.05, pseudo *R*^2^ = 0.88). However, none of the factors investigated were identified as independent indicators of these performance criteria (scope of practice, all *P* > 0.10, autonomy, all *P* > 0.15).

## Discussion

This study sought to establish the demographics, scope of practice, autonomy and training of UK hospital optometrists involved in the provision of ophthalmic laser procedures. The results strongly suggest that hospital optometrists perform ophthalmic laser procedures across the UK. Activity predominantly relates to glaucoma and cataract care. This reflects the scope of practice for which the UK Ophthalmology Alliance have produced policy guidance and aligns with the areas that were introduced to UK optometrists from external sources such as the Northeastern State University, Oklahoma College of Optometry in 2021 [26–28].

In this survey, 91% of hospital laser-optometrists reported that they made the clinical decision to treat independently and autonomously. This finding has important ramifications for eye care guidance in the UK. In alignment with the results, the National Institute for Health and Care Excellence (NICE) glaucoma NG81 guideline recognises that the upregulation of SLT will necessitate a change to the organisation of glaucoma care [7]. Conversely, the present findings indicated little adherence to the NICE recommendation for healthcare professionals to discuss the decision to offer 360° SLT and how it will be performed with the responsible consultant ophthalmologist [7]. In conjunction with this observation, alongside studies that have evidenced the safety of optometrists performing laser procedures [15–18, 29], optometric associations and insurers should also consider updating their policies accordingly.

Autonomous management of any condition in healthcare should always be based on a combination of ethical, legal and operational rules that govern how decisions are made, particularly concerning patient care. To facilitate this, the College of Optometrists provides clinical management guidelines and higher qualifications, which aim to provide a framework for safe, evidence-based optometric practice for UK optometrists. Presently, the College of Optometrists Professional Diploma in Glaucoma enables optometrists to manage or co-manage patients with established glaucoma autonomously [30]. The learning outcomes specify the need for an understanding of the indications for, techniques used, expected outcomes and complications of laser therapies used in the management of glaucoma. Based on the outcomes reported, this should be updated to reflect the role hospital optometrists already have in autonomous treatment of glaucoma using ophthalmic laser procedures.

The results of this study reflect the trend found by other hospital optometrist surveys that have reported growth both in terms of size and scope of practice [13]. This might be underpinned by the national move towards using more allied healthcare professionals to deliver treatments and work at the top of their respective clinical license [31–34]. It might also reflect increased professional interest in this area, which has led to the establishment of educational programmes to train optometrists in laser procedures [22–25]. In addition, the January 2022 update to the NICE glaucoma diagnosis and management guideline, advocating 360° SLT as an initial treatment and/or repeat treatment under certain conditions for those with OHT and COAG, will likely have upregulated laser activity in some areas, resulting in increased allied healthcare involvement to reinforce service delivery [7]. A decade ago, areas such as glaucoma monitoring were typically considered an extended role for hospital optometrists. Presently, involvement in glaucoma clinics is far more commonplace and often a core hospital optometrist activity [13]. Expansion of hospital optometrist activity into other extended roles such as uveitis, vitreoretinal services and neuro ophthalmology has also been reported [13]. The growing trend in the number of hospital optometrists practising new procedures and managing more complex patients autonomously clearly aligns with the increased involvement in ophthalmic laser procedures reported in the present study.

Regional variations in activity were found; the results did not include any responses from hospital laser-optometrists based in the East Midlands and Northern Ireland. This is in contrast to the surrounding areas. Despite this, it is not sensible to imply inadequacy of service in these regions. Although it is possible that at the time of study, no optometrists in these areas were delivering ophthalmic laser treatments. It is also feasible that optometrists are laser active in these regions, but are situated in commissioned service pathways outside the traditional NHS HES setting [31, 32]. Similarly, it may simply reflect a regional lack of engagement with the survey. In addition, when considering the distribution of hospital laser-optometrists, the present study cannot determine competency equivalency. Given the multiple, varying paths available to trainee hospital laser-optometrists, this is an important consideration.

Those who had been qualified longer tended to have been involved in ophthalmic laser procedures for a greater period. However, representation across a wide range of years qualified was found in response to the survey. In addition, years qualified and duration performing ophthalmic laser procedures were not independent predictors of the scope of ophthalmic laser practice or level of autonomy. In alignment with this and the wide range of involvement reported, the results do not support a minimum qualifying period for hospital optometrists before initiating ophthalmic laser training. Therefore, further consideration may be given to the depth of education and training opportunities provided relating to laser treatment within university optometry programmes.

Although independent prescriber status existed within the cohort, not all respondents were independent prescribers. Considering the potential need for pre/post ocular therapeutics, clinicians who engage in laser provision require access to necessary drugs; therefore, it is likely that respondents who were predominantly autonomous in their laser provision, yet are not registered as an independent prescriber, are reliant on patient group directions or similar local protocols to facilitate the prescription and supply of ocular drugs.

Given the notable rise in the number of optometrists with the College of Optometrists’ higher qualifications from 2021 to 2024, it is clear that although these qualifications are not a requirement for involvement in ophthalmic laser provision, optometrists are willing to upskill to gain qualifications in clinical subspecialties [35]. Despite this, training pathways are still largely based in-house, despite an emergence of optometrists attending courses by universities or other independent providers. Invariably, internal hospital training programmes may vary in content and length. The results of the present survey indicate that the assessment strategy typically requires a trainee to treat a predefined number of cases and/or to demonstrate their clinical skillset under direct observation. The use of simulation eyes was uncommon, suggesting that these training aids are more likely to be encountered in courses hosted outside the hospital environment, where access to patients requiring laser treatment is limited.

Based on the traditional model of patient care in consultant-led face-to-face clinics, the care for all patients whose condition falls outside the autonomous competencies of the examining practitioner is deemed the responsibility of a consultant ophthalmologist and should be risk assessed. This means that the final decision relating to the involvement of staff in care provision currently depends on a supervising consultant ophthalmologist’s discretion. The level of autonomy reported in the present study suggests that post-training, the degree of direct oversight for hospital optometrists involved in the most commonly performed laser procedures in this study is low. This outcome supports the acceptance of existing training procedures despite their variability. This is likely because training is most often conducted in-house, under the remit of resident senior clinicians. In comparison, the wider acceptance of external training is yet to be determined.

It is widely recognised that the current model of care in UK hospitals for healthcare practitioners is variable. The scope of involvement in patient care across subspecialties ranges from some healthcare practitioners being utilised as data collectors with all their cases reviewed to validate their preliminary decision-making, to those in the present study who are predominantly independent and autonomous in their decision-making. To ensure clinic capacity and practitioner skill use is optimised, flexibility should be advocated. For example, as long as eye care is provided within a protocol-driven framework of clinical governance, audit and systematic quality control guidance should not reflect the location or service delivery pathway or type, but rather empower optometrists to continue to diagnose and treat their patients safely using ophthalmic lasers where appropriate, both within the HES and, where local facilities allow, in community eye care settings. Developing communication channels and improving the flow of information between optometrists and ophthalmologists would greatly benefit both professions to implement such a strategy.

### Limitations and Future Work

Although there was only a small number of respondents, in accordance with the latest estimate, this is likely representative of the wider UK cohort. However, by virtue of the methodology used, this study is susceptible to selection bias. Distribution of the survey via professional networks and social media channels may favour practitioners who are highly autonomous and/or glaucoma-focused. In alignment with this, as more hospital optometrists with an involvement in glaucoma care responded than any other subspecialty, the results are also less translatable to subspecialties outside this area.

The subjective nature of this study is also a consideration, as it is for all questionnaire data. It is not possible to discount that a respondent returned a completed survey more than once. Albeit this is an unlikely occurrence, to mitigate repeat responses, participants were informed via a preamble not to complete the survey more than once or via multiple channels. Post hoc review of the data showed no duplicate entries. Mapping years qualified, hours involved in laser provision and location also yielded no directly overlapping input from respondents.

Finally, it cannot be guaranteed that only hospital optometrists completed the survey, as opposed to those who perform laser procedures within the private sector. The average number of hours per week (30) working within an HES is suggestive that the responses received are predominantly from the correct cohort. Exploring optometrist laser provision within the private sector and involvement of other allied healthcare practitioners working within secondary care have been identified as areas of future work.

## Conclusion

Hospital optometrists provide patient-centred laser eye care across the UK. This is important given the continued pressures on NHS ophthalmology services. Evidence supports comparable optometrist-ophthalmologist safety profiles and clinical outcomes for SLT, one of the most commonly performed laser procedures identified in this study [15–18]. In addition, optometrists within the glaucoma subspecialty currently make the decision to treat independently and autonomously. This outcome is not well aligned with current national glaucoma guidance.

As this survey shows that optometrists provide laser procedures autonomously, guidance should be updated to better reflect this current practice. Continued strengthening of the relationship between optometry and ophthalmology should also be encouraged to nurture and expand this beneficial trend for patient care.

## Supplementary Information

Supplement 1

## Data Availability

Data available from the corresponding author via reasonable request.

## References

[1] World Council of Optometry. WCO’s Concept of Optometry. St Louis (USA): World Council of Optometry; 2025 [reviewed 2021; cited 2025 Jul 23]. Available from: https://worldcouncilofoptometry.info/concept-of-optometry/.

[2] General Optical Council. Rules relating to injury of disease of the eye order of council. London (UK): UK Statutory Instruments No 3267; 1999 [cited 2025 Jul 23]. Available from: https://www.legislation.gov.uk/uksi/1999/3267.

[3] Crown J. Review of prescribing, supply and administration of medicines. London (UK): HSC Public Health Agency; 1999 [cited 2025 Jul 23]. Available from: https://www.publichealth.hscni.net/sites/default/files/directorates/files/Review%20of%20prescribing%2C%20supply%20and%20administration%20of%20medicines.pdf.

[4] General Optical Council. Injury or disease of the eye and contact lens (qualifications) amendment rules order of council. London (UK): UK Statutory Instruments No 1476; 2005 [cited 2025 Jul 23]. Available from: https://www.legislation.gov.uk/uksi/2005/1476/made.

[5] General Optical Council. Therapeutics and contact lens specialties rules order of council. London (UK): UK Statutory Instruments No 1940; 2008 [cited 2025 Jul 23]. Available from: https://www.legislation.gov.uk/uksi/2008/1940/made.

[6] Medicines and Healthcare Products Regulatory Agency. The Human Medicines Regulations. London (UK): UK Statutory Instruments No 1916; 2012 [cited 2025 Jul 23]. Available from: https://www.legislation.gov.uk/uksi/2012/1916/contents.

[7] National Institute for Health and Care Excellence. Glaucoma: diagnosis and management. London (UK): NICE guideline NG81; 2017 [reviewed 2022; cited 2025 Jul 23]. Available from: https://www.nice.org.uk/guidance/ng8. 31909934

[8] The Royal College of Ophthalmologists. Facing workforce shortages and backlogs in the aftermath of COVID-19: the 2022 census of ophthalmology consultant, trainee and SAS workforce. London (UK): The Royal College of Ophthalmologists; 2023 [cited 2025 Aug 5]. Available from: https://www.rcophth.ac.uk/wp-content/uploads/2023/03/2022-Ophthalmology-census-Facing-workforce-shortages-and-backlogs-in-the-aftermath-of-COVID-19.pdf.

[9] The College of Optometrists. Clinical Council for Eye Health Commissioning. London (UK): The College of Optometrists; 2025 [cited 2025 Jul 23]. Available from: https://www.college-optometrists.org/clinical-council-for-eye-health-commissioning.

[10] Court H, Dougall J, Pooley J. The community optometry workforce in Scotland: supporting sustainable eye care delivery. Eye. 2025;39:1003–8. 10.1038/s41433-024-03573-5. 39827238 10.1038/s41433-024-03573-5 PMC11933349

[11] Harper R, Edgar DF, Parkins DJ. Shifting left for getting it right: Lessons from primary care optometry developments in Scotland. Eye. 2025;39:804–5. 10.1038/s41433-025-03620-9. 39863705 10.1038/s41433-025-03620-9 PMC11933453

[12] The College of Optometrists. UK Eye Care Data Hub: forecasting eye health and workforce needs. London (UK): York Health Economics Consortium 2025 [cited 2025 Jul 23]. Available from: https://ukeyecaredatahub.shinyapps.io/UK_Eye_Care_Data_Hub/.

[13] Gunn PJG, Creer RC, Bowen M, Tromans C, Jackson AJ, Tompkin AP, et al. Scope of practice of optometrists working in the UK Hospital Eye Service: Second national survey. Ophthalmic Physiol Opt. 2022;42:428–39. 10.1111/opo.12952. 35150447 10.1111/opo.12952 PMC9303216

[14] Harper R, Creer R, Jackson J, Ehrlich D, Tompkin A, Bowen M, et al. Scope of practice of optometrists working in the UK Hospital Eye Service: a national survey. Ophthalmic Physiol Opt. 2016;36:197–206. 10.1111/opo.12262. 26555386 10.1111/opo.12262

[15] Lee CN, Delaney A, Richardson JAL, Freeman G, Gunn PJG, Harthan S, et al. Comparative outcomes of selective laser trabeculoplasty delivered by optometrists compared with ophthalmologists: a UK-based multicentre observational study. BMJ Open Ophthalmol. 2024;9:e001870. 10.1136/bmjophth-2024-001870. 39357974 10.1136/bmjophth-2024-001870 PMC11448136

[16] Chadwick O, Chia SN, Rotchford A. Establishing an allied health professional delivered selective laser trabeculoplasty service in Scotland. Ophthalmic Physiol Opt. 2019;39:216–23. 10.1111/opo.12611. 30994202 10.1111/opo.12611

[17] Konstantakopoulou E, Varia J, Parmar J, Nathwani N, Hau S, Low WS, et al. Optometrist-delivered selective laser trabeculoplasty in the HES—a training protocol and early service evaluation. Eye. 2024;38:2589–95. 10.1038/s41433-024-03086-1. 38702512 10.1038/s41433-024-03086-1 PMC11385571

[18] Jones L, Konstantakopoulou E, Gazzard G. Selective laser trabeculoplasty (SLT) performed by optometrists for patients with glaucoma and ocular hypertension: a scoping review protocol. BMJ Open Ophthalmol. 2020;5:e000438. 10.1136/bmjophth-2020-000438. 32509963 10.1136/bmjophth-2020-000438 PMC7252989

[19] Konstantakopoulou E, Jones L, Nathwani N, Gazzard G. Selective laser trabeculoplasty (SLT) performed by optometrists-enablers and barriers to a shift in service delivery. Eye. 2022;36:2006–12. 10.1038/s41433-021-01746-0. 34389819 10.1038/s41433-021-01746-0 PMC8362647

[20] Stein JD, Zhao PY, Andrews C, Skuta GL. Comparison of outcomes of laser trabeculoplasty performed by optometrists vs ophthalmologists in Oklahoma. JAMA Ophthalmol. 2016;134:1095–101. 10.1001/jamaophthalmol.2016.2495. 27467233 10.1001/jamaophthalmol.2016.2495

[21] The UK Ophthalmology Alliance. N:d YAG laser capsulotomy by nurses and optometrists policy and procedure. London (UK): Moorfields Eye Hospital; 2018 [cited 2025 Jul 23]. Available from: https://uk-oa.co.uk/wp-content/uploads/2018/07/UKOA_Worksteams_Extended_Roles_nd_yag_laser_capsulotomy_by_nurses_and_optometrists_v3_0.pdf.

[22] Moorfields Education. Laser course for ophthalmic healthcare professionals. London (UK): Moorfields Education; 2025 [cited 2025 Jul 23]. Available from: https://checkout.moorfields.nhs.uk/product?catalog=Laser-course-for-ophthalmic-healthcare-professionals.

[23] Cardiff University. OPT043 ophthalmic therapeutic laser procedures—foundation. Cardiff (UK): Cardiff University School of Optometry and Vision Sciences; 2025 [cited 2025 Jul 23]. Available from: https://www.cardiff.ac.uk/optometry-vision-sciences/courses/postgraduate-taught/modules/opt043-ophthalmic-therapeutic-laser-procedures-foundation.

[24] Aston University. Advanced optometry and ophthalmology clinical skills workshop. Birmingham (UK): Aston University College of Health and Life Sciences; 2025 [cited 2025 Jul 23]. Available from: https://www.aston.ac.uk/hls/advanced-optometry-and-ophthalmology-clinical-skills-workshop.

[25] Lighthizer N, Patel K, Cockrell D, Leung S, Harle DE, Varia J, et al. Establishment and review of educational programs to train optometrists in laser procedures and injections. Clin Exp Optom. 2025;108:248–57. 10.1080/08164622.2024.2380075. 39048296 10.1080/08164622.2024.2380075

[26] The UK Ophthalmology Alliance. Clinical practice pack for non-medical practitioners: YAG laser capsulotomy. London (UK): The UK Ophthalmology Alliance; 2019 [cited 2025 Aug 5]. Available from: https://www.uk-oa.co.uk/wp-content/uploads/2020/03/UKOA_laser_YAG_Caps_policy_Oct-2019-2.pdf.

[27] The UK Ophthalmology Alliance. Clinical practice pack for non-medical practitioners: Laser treatment in the glaucoma service. London (UK): The UK Ophthalmology Alliance; 2019 [cited 2025 Aug 5]. Available from: https://uk-oa.co.uk/wp-content/uploads/2020/03/UKOA-SLT-and-PI-laser-practice-pack_Oct-2019.docx.pdf.

[28] Northeastern State University. Ophthalmic procedures course. Oklahoma (USA): Oklahoma College of Optometry; 2025 [cited 2025 Aug 5]. Available from: https://optometry.nsuok.edu/continuingeducation/Ophthalmic_Procedures.aspx.

[29] Swystun AG, Burton D, Edwards A, Alaghband P. The safety and effectiveness of optometrist delivered laser peripheral iridotomy. Eye. 2026;5:512-4. 10.1038/s41433-025-04207-0. 10.1038/s41433-025-04207-0 PMC12957287 41495442

[30] The College of Optometrists. Programme to prepare optometrists to manage or co-manage patients with established glaucoma (diploma level). London (UK): The College of Optometrists; 2015 [cited 2026 Feb 20]. Available from: https://www.college-optometrists.org/coo/media/media/documents/higher%20qualifications/learning-outcomes-diploma-in-glaucoma.pdf.

[31] National Health Service England. Neighbourhood health guidelines 2025/26. London (UK): NHS Community Health Services; 2025 [cited 2025 Aug 5]. Available from: https://www.england.nhs.uk/long-read/neighbourhood-health-guidelines-2025-26/.

[32] National Health Service England. Working to maximise the contribution of allied health professions. London (UK): NHS Workforce, Training and Education; 2025 [cited 2025 Aug 5]. Available from: https://www.hee.nhs.uk/our-work/allied-health-professions/working-maximise-contribution-allied-health-professions.

[33] National Health Service Wales. NHS Wales eye health care future approach to optometry services. Cardiff (UK): Welsh Government; 2021 [cited 2025 Aug 5]. Available from: https://www.gov.wales/sites/default/files/publications/2021-03/nhs-wales-eye-health-care-future-approach-for-optometry-services.pdf.

[34] Optometry Scotland. Advancing world-class eye care in Scotland: strategy 2024-2027. Edinburgh (UK): The Scottish Parliament; 2024 [cited 2025 Aug 5]. Available from: https://www.parliament.scot/-/media/files/committees/health-social-care-and-sport-committee/correspondence/2024/optometry-scotland-strategy-launch.pdf.

[35] Ellek N, Gunn PJ, Bowen M, Harper RA. Mapping glaucoma higher qualifications within UK optometry. Ophthalmic Physiol Opt. 2025;45:1221–7. 10.1111/opo.13502. 40192457 10.1111/opo.13502

