# Supplementary material for: A Survey of Hospital Optometrists Performing Ophthalmic Laser Procedures in the United Kingdom
Source: Ophthalmic Physiol Opt. 2026 May 19;46(4):952–8. doi: 10.1007/s44402-026-00107-2 (PMC13395820; doi:10.1007/s44402-026-00107-2)
Supplement: Supplementary file 1 — Supplement 1 [file 44402_2026_107_MOESM1_ESM.docx]

**Suppl 1** Digital questionnaire

**SECTION 1. DEMOGRAPHICS**

1. **How long have you been a qualified optometrist?**
2. 0-5 years
3. 6-10 years
4. 11-15 years
5. 16-20 years
6. Over 20 years
7. **Where is the hospital or secondary care environment you usually work in?**
8. England – London
9. England – North East
10. England – North West
11. England – Yorkshire and The Humber
12. England – East Midlands
13. England – West Midlands
14. England – East of England
15. England – South East
16. England – South West
17. Wales
18. Scotland
19. Northern Ireland
20. **How many hours per week do you typically work in a hospital or secondary care environment?**

- Enter number

1. **How many hours per week do you typically perform ophthalmic laser procedures?**

- Enter number

1. **How long ago did you start performing ophthalmic laser procedure(s)?**
2. Less than 1 year
3. 2-3 years
4. 4-5 years
5. 5-10 years
6. Over 10 years
7. **Are you an independent prescriber?**
   1. Yes
   2. No

**SECTION 2. APPLICATION AND TRAINING**

1. **Which laser procedure(s) are you currently performing?** Tick all that apply
2. Photorefractive keratectomy
3. Laser in situ keratomileusis
4. Small incision lenticule extraction
5. Femtosecond laser in cornea
6. Argon laser trabeculoplasty
7. Selective laser trabeculoplasty
8. Micropulse laser trabeculoplasty
9. Peripheral laser iridotomy
10. Laser Iridoplasty
11. Endoscopic cytophotocoagulation
12. Femtolaser assisted cataract surgery
13. Nd:YAG capsulotomy
14. Laser vitreolysis
15. Pan retinal photocoagulation
16. Focal and grid macular laser treatment
17. Laser retinopexy
18. Other – Please specify
19. **Which laser procedure(s), that you do not currently perform, may you start performing in the near future?** Tick all that apply, leave blank if no plans to expand your current scope of ophthalmic laser procedure work in the near future.
20. Photorefractive keratectomy
21. Laser in situ keratomileusis
22. Small incision lenticule extraction
23. Femtosecond laser in cornea
24. Argon laser trabeculoplasty
25. Selective laser trabeculoplasty
26. Micropulse laser trabeculoplasty
27. Peripheral laser iridotomy
28. Laser Iridoplasty
29. Endoscopic cytophotocoagulation
30. Femtolaser assisted cataract surgery
31. Nd:YAG capsulotomy
32. Laser vitreolysis
33. Pan retinal photocoagulation
34. Focal and grid macular laser treatment
35. Laser retinopexy
36. Other – Please specify
37. **When performing ophthalmic laser procedures do you predominantly:**
38. Work independently and autonomously making the clinical decision to treat
39. Work independently and autonomously performing treatments after an ophthalmologist has made the clinical decision to treat
40. Work under direct supervision without autonomy performing treatments after an ophthalmologist has made the clinical decision to treat
41. Other – please specify
42. **Was your ophthalmic laser procedures training conducted by a UK institution or one outside the UK?**
    1. Yes
    2. No

- If No: Which non-UK Institution was your initial ophthalmic laser procedures training conducted?

1. **Which best describes the training you received in ophthalmic laser procedures**
2. Formal training from a higher education Institution e.g. University
3. Formal training from an independent provider not associated with a higher education institution
4. In-house training within the hospital
5. Other – please specify
6. **Did you practice performing ophthalmic laser procedures on simulation eyes at any point during your training period?**
   1. Yes
   2. No
7. **Which best describes the benchmark for completion of the training period?** Please tick all that apply
   1. I had to pass a theory assessment
   2. I had to pass a formal practical assessment
   3. I had to demonstrate my clinical skillset in a non-formal assessment setting
   4. I had to observe a predefined number of patient cases
   5. I had to treat a predefined number of patient cases
   6. Other – Please specify
